# Longitudinal Correlations Between Intravoxel Incoherent Motion (IVIM) and Dynamic Contrast-Enhanced (DCE) MRI During Radiotherapy in Prostate Cancer Patients

**DOI:** 10.3389/fonc.2022.897130

**Published:** 2022-06-07

**Authors:** Ernst S. Kooreman, Vivian van Pelt, Marlies E. Nowee, Floris Pos, Uulke A. van der Heide, Petra J. van Houdt

**Affiliations:** Department of Radiation Oncology, The Netherlands Cancer Institute, Amsterdam, Netherlands

**Keywords:** DCE, IVIM, prostate cancer, treatment response, repeated measures, correlations, perfusion

## Abstract

**Purpose:**

Intravoxel incoherent motion (IVIM) is a promising technique that can acquire perfusion information without the use of contrast agent, contrary to the more established dynamic contrast-enhanced (DCE) technique. This is of interest for treatment response monitoring, where patients can be imaged on each treatment fraction. In this study, longitudinal correlations between IVIM- and DCE parameters were assessed in prostate cancer patients receiving radiation treatment.

**Materials and Methods:**

20 prostate cancer patients were treated on a 1.5 T MR-linac with 20 x 3 or 3.1 Gy. Weekly IVIM and DCE scans were acquired. Tumors, the peripheral zone (PZ), and the transition zone (TZ) were delineated on a T_2_-weighted scan acquired on the first fraction. IVIM and DCE scans were registered to this scan and the delineations were propagated. Median values from these delineations were used for further analysis. The IVIM parameters D, f, D^*^ and the product fD^*^ were calculated. The Tofts model was used to calculate the DCE parameters K^trans^, k_ep_ and v_e_. Pearson correlations were calculated for the IVIM and DCE parameters on values from the first fraction for each region of interest (ROI). For longitudinal analysis, the repeated measures correlation coefficient was used to determine correlations between IVIM and DCE parameters in each ROI.

**Results:**

When averaging over patients, an increase during treatment in all IVIM and DCE parameters was observed in all ROIs, except for D in the PZ and TZ. No significant Pearson correlations were found between any pair of IVIM and DCE parameters measured on the first fraction. Significant but low longitudinal correlations were found for some combinations of IVIM and DCE parameters in the PZ and TZ, while no significant longitudinal correlations were found in the tumor. Notably in the TZ, for both f and fD^*^, significant longitudinal correlations with all DCE parameters were found.

**Conclusions:**

The increase in IVIM- and DCE parameters when averaging over patients indicates a measurable response to radiation treatment with both techniques. Although low, significant longitudinal correlations were found which suggests that IVIM could potentially be used as an alternative to DCE for treatment response monitoring.

## 1 Introduction

Non-invasive perfusion imaging is of interest in oncology, as low perfusion is related to hypoxia which holds prognostic value (1–3). A common way to measure perfusion is by using dynamic contrast enhanced (DCE-) MRI (2, 4, 5). In addition to prognosis, DCE has been shown to have value for mid-treatment response assessment in cervix (6), esophageal (7), and head-and-neck cancer (8–10).

Acquiring quantitative MRI (qMRI) images during radiation treatment for the purpose of treatment response monitoring has become feasible with the introduction of MR-guided radiotherapy. Using MR-linacs, which consist of a linear accelerator integrated with an MRI system, qMRI sequences can be acquired on each treatment fraction, without the increase of patient burden (11–16).

Although DCE-MRI is a candidate for treatment response monitoring, acquiring a DCE scan during each treatment fraction is undesirable due to the use of contrast agent. Alternative techniques that can provide perfusion information without the use of contrast agent are needed. One such alternative is intravoxel incoherent motion (IVIM), which is an extension to diffusion weighted imaging (DWI) (17). IVIM parameters provide information about diffusion and perfusion. It is based on the concept that inside a voxel, signal from water flowing in the capillaries can be separated from diffusing water (18). In addition to the diffusion coefficient (D), the perfusion parameters f (perfusion fraction), D^*^ (pseudo-diffusion coefficient), and the product fD^*^ can be determined.

Previous studies have investigated correlations between IVIM and DCE-MRI parameters in different tumor sites, with conflicting results (19). These studies usually determine the correlation between IVIM and DCE parameters on a single time point. For treatment response purposes however, correlations between changes in parameters, induced by radiation treatment, are more relevant. A study performed in 21 liver tumor-bearing rabbits assessed the correlations between IVIM and DCE parameters longitudinally, while the rabbits were treated with a vascular disrupting agent (20). Interestingly, the authors did not find any significant correlations between IVIM and DCE parameters when assessing the imaging time points separately, but did find a significant longitudinal correlation. This longitudinal correlation is of importance for treatment response monitoring purposes and indicates that IVIM could be a potential substitute for DCE-MRI for this purpose.

In the current study, longitudinal correlations between IVIM- and DCE parameters are assessed in a cohort of prostate cancer patients that were imaged weekly during radiation treatment. Each week a DCE and an IVIM scan were acquired to enable longitudinal assessment. The aim of this study is to determine whether IVIM and DCE parameters correlate when measured longitudinally and whether there is potential for IVIM to substitute DCE for treatment response monitoring.

## 2 Materials and Methods

### 2.1 Patients

Twenty patients, with a median age of 70.5 (range 53 – 82) years with biopsy proven prostate cancer were included in this study. Only patients with an adequate renal function (glomerular filatration rate GFR > 60 ml/min/1.7m^2^) were included. Thirteen patients were treated with 20 x 3 Gy and due to a change in clinical practice, seven patients were treated with 20 x 3.1 Gy. Treatment took place over the course of five weeks. Patient characteristics are presented in **Table 1**. The study was approved by the local ethics committee and each patient gave written informed consent.

**Table 1:** Patient characteristics.

Patient characteristic	Median (range)
Age (years)	70.5 (53 – 82)
iPSA (ng/ml)	15 (8 – 38)
GFR (ml/min/1.7m^2^)	
Pre-treatment	79 (67 – 107)
Post-treatment	82 (65 – 110)
ISUP	No. of patients
1	3
2	8
3	4
4	3
5	2

iPSA, initial prostate specific antigen; GFR, glomerular filtration rate; ISUP score, prostate cancer grading score.

### 2.2 Image Acquisition

All patients were treated on a 1.5 T MR-linac (Unity, Elekta AB, Stockholm, Sweden). This is a hybrid system, where a linear accelerator is integrated with an MRI scanner to enable concurrent patient irradiation and MRI acquisition. The MRI system of the MR-linac is based on a 1.5 T Ingenia system (Philips Healthcare, Best, The Netherlands), with split gradient coils to create a window for the radiotherapy beam (21). The system uses an 8-channel radio-translucent phased array receive coil (22).

A T_2_-weighted anatomical scan, an IVIM scan and a DCE-MRI scan were acquired weekly over the course of five weeks, starting at the first day of treatment. Scan parameters can be found in **Table 2**. The IVIM sequence was optimized for the MR-linac system, which has lower gradient performance compared to diagnostic systems and lower SNR due to the simpler receive coil system (15, 23). To compensate this, the highest b-value was limited to 500 s/mm^2^, and a relatively large isotropic acquisition voxel size of 4 mm^3^ was used. To calculate contrast agent concentration values, the pre-contrast T_1_ was measured using the variable flip angle (VFA) method with a similar sequence as the DCE scan, but with a TR/TE of 20/4 ms and flip angles of 3, 6, 10, 20, and 30 ˚. For the DCE scan, during the fifth dynamic, 15 mmol gadoteric acid (Dotarem, Geurbet, France) was injected at a rate of 3 mL/s using a power injector followed by a 30 ml saline flush. While a study by Wang et al. demonstrated no significant effect of radiation on the chemical composition of Gadolinium based contrast agents (24), DCE scans were acquired after the radiation treatment, without repositioning of the patient to avoid interactions of the contrast agent with radiation.

**Table 2:** MRI sequence parameters.

	T_2_-weighted	IVIM	DCE
Sequence type	3D-TSE	ss-EPI	3D-FFE
Field of view (mm^3^)	400 x 448 x 250	430 x 430 x 60	220 x 251 x 60
Acquired voxel size (mm^3^)	1.2 x 1.2 x 1.2	3.98 x 3.98 x 4.00	2.62 x 2.62 x 7.00
Reconstructed voxel size (mm^3^)	0.57 x 0.57 x 1.2	1.92 x 1.92 x 4.00	1.57 x 1.57 x 3.50
Flip angle (°)	90	90	35
TR/TE (ms)	1300/129	2960/82	4.0/1.9
Fat suppression	–	SPAIR	–
Parallel imaging (SENSE) factor	3.5	2.3	2
Acceleration factor	110	47	–
b-values (averages) (s/mm^2^)	–	0 (8), 30 (8), 150 (8), 500 (16)	–
Phase encoding bandwidth (Hz/pixel)	–	32.9	–
Gradient timings Δ/δ (ms)	–	41.1/20.0	–
Dynamic scan time (s)	–	–	2.8
Number of dynamics	–	–	110
NSA	2	1	1
Acquisition time (m:ss)	5:48	5:11	5:04

### 2.3 Image Registration

Tumor, peripheral zone (PZ), and transition zone (TZ) were delineated on the T_2_-weighted scans of the first fraction. Of three patients, who received a trans-urethral resection of the prostate (TURP), the TURP cavity was delineated to be excluded from analysis. Tumors were delineated while consulting biopsy results and diagnostic images, following the PI-RADS V2.1 criteria (25).

The IVIM and DCE images were registered separately to the T_2_-weighted scan of the first fraction which contained the delineations using rigid registration allowing rotations and translations. For IVIM, the b = 0 s/mm^2^ image was used as this contains the most anatomical information. For DCE, the 100^th^ dynamic was used as a scan with relatively high enhancement in the prostate signal. All registrations were checked visually and corrected manually when needed. After registration, the delineations were propagated to the IVIM and DCE scans, where only voxels that were fully inside the propagated delineation were included for further analysis.

IVIM scans were excluded when susceptibility artifacts were present inside any of the delineations, or when movement between b-values was present. DCE scans were excluded if patient movement occurred during the scan.

The volume of the structures was calculated by multiplying the number of voxels completely inside the delineation by the voxel size of the T_2_-weighted scan they were delineated on.

### 2.4 Image Processing

#### 2.4.1 IVIM

The bi-exponential IVIM model, S_(b)_ / S_0_ = fe ^–bD*^ + (1 - f) e ^-bD^, was fitted using a segmented approach (26). Using the median signal intensity values from the delineations, the tissue diffusion coefficient (D) was determined first using the two highest b-values (150 and 500 s/mm^2^). Next the perfusion fraction (f) was calculated using this D and the b = 0 s/mm^2^ signal intensity. Both D and f were then used in combination with the signal intensities from the lowest two b-value images (0 and 30 s/mm^2^) to calculate the pseudo-diffusion coefficient D^*^. The parameter fD^*^ was calculated by multiplying f with D^*^.

#### 2.4.2 DCE

To extract an arterial input function (AIF), external iliac artery was delineated on all DCE scans of all patients. Due to slight variations in the B_1_ field (see **Supplementary Figure 1**), only the left external iliac artery was used. Signal intensities were converted to concentration time curves using the spoiled gradient echo equation following Schabel and Parker (27) assuming a T_1_ value of 1429 ms for blood at 1.5 T (28) and a contrast agent relaxivity of 3.6 L mM^-1^ s^-1^ (29). Following Georgiou et al. the maximum relative change in concentration during the DCE scan was determined for all voxels inside this delineation (30). The voxels between the 50^th^ and 95^th^ percentile of this relative change were averaged to obtain an AIF for each treatment fraction. Per patient, the median AIF of all five measurements, based on peak height, was used for all tracer kinetic modeling for that patient. **Supplementary Figure 2** shows all AIFs of all patients.

A voxel-wise T_1_-map was calculated from the VFA series using a linear implementation (31). The T_1_ map was used to convert signal intensity to concentration values using the method of Schabel and Parker (27). The bolus arrival time was estimated for each voxel using an automated method (32). The volume transfer constant (K^trans^) and the rate constant (k_ep_) from the standard Tofts model (33) were calculated on a voxel-basis following the approach developed by Murase (34) using and median AIF as input. The extracellular extravascular space volume fraction (v_e_) was then calculated on a voxel basis using (K^trans^/k_ep_).

### 2.5 Statistics

Baseline values from the IVIM and DCE parameters were taken from the scans of the first fraction. To check for differences in parameters between ROIs, a one-way analysis of variance (ANOVA) was performed for each parameter, with ROI as the independent variable. ANOVA results are presented with their *F*-statistic including within- and between group degrees of freedom, and *p*-value. Pearson correlation coefficients were calculated between IVIM and DCE parameters of the first fraction for each ROI.

To determine longitudinal correlations between the IVIM and DCE parameters, the *rmcorr* package in R was used (35). The rmcorr package provides a repeated measures correlation (*r_rm_*), which takes into account the non-independence of repeated measures. To do so, the relationship between two continuous variables (in this case the IVIM and DCE parameters) is determined while controlling for between-patient variance. Specifically, separate parallel lines are fitted to the data of each patient using a common slope but allowing the intercept to vary per patient (35). The *r_rm_* is then calculated from the sum of squares values for the measure and the error as follows:


$$r_{rm}=\sqrt{\frac{{\text{SS}}_{\text{Measure}}}{{\text{SS}}_{\text{Measure}}+{\text{SS}}_{\text{Error}}}}$$

The sign of *r_rm_* is taken from the sign of the common slope. The degrees of freedom are calculated using N(k-1)-1, where k is the (average) number of repeated measures per participant and N is the total number of participants (35).

As IVIM and DCE measure different biological properties which are both related to perfusion, it is possible that their correlation depends on the particular tissue measured. Therefore, *r_rm_* was calculated separately for each ROI. It can be interpreted as the intra-patient correlation between IVIM and DCE parameters during radiation treatment for a given ROI. Repeated measures correlation results are presented as *r_rm_* (error degrees of freedom), *p*-value, and a 95% confidence interval calculated using bootstrapping with 10.000 resamples. Statistical significance was assumed for all tests when *p* < 0.05.

## 3 Results

Imaging data was acquired on five fractions for 19/20 patients and one patient was imaged four times. This resulted in a total of 99 fractions with IVIM and DCE scans. Two DCE scans were excluded due to movement during acquisition, both from the same patient. Seven IVIM scans were excluded due to susceptibility artifacts causing deformations within the delineations and two IVIM scans were excluded because the patient moved between the acquisition of images with a different b-value, leaving 97 DCE acquisitions and 90 IVIM acquisitions for further analysis.

**Figure 1:**
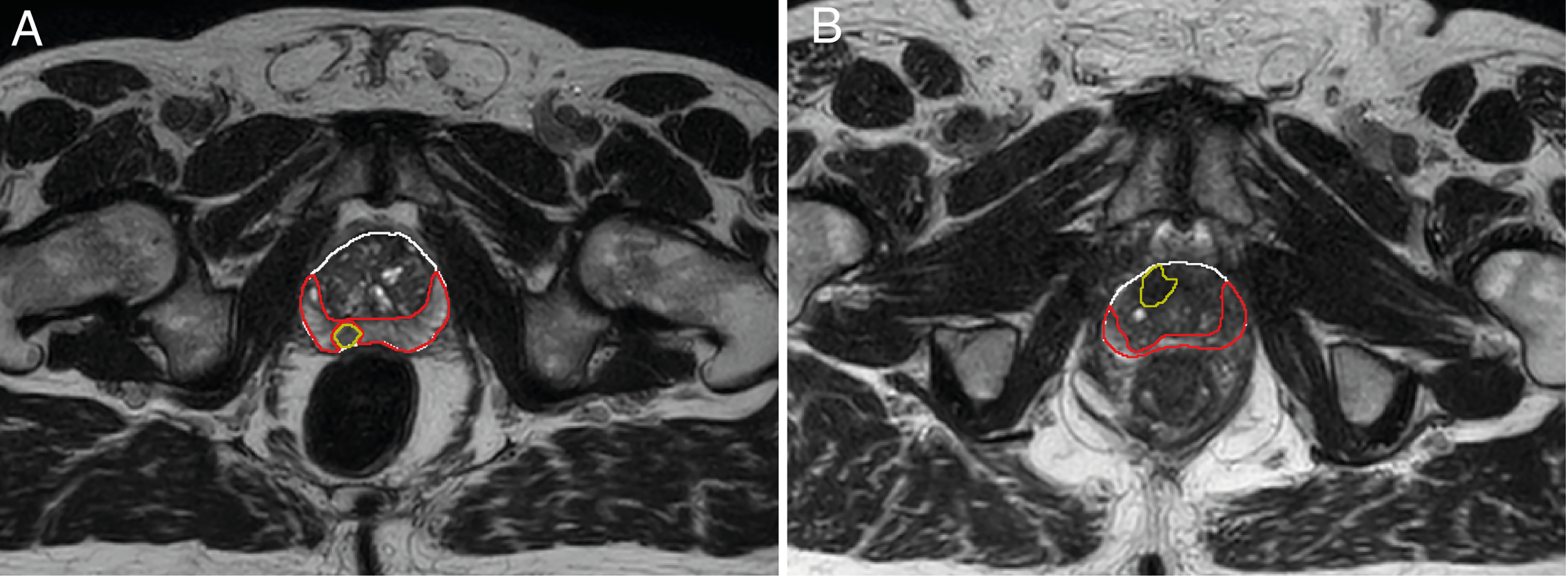
Example of delineations of the different prostate zones of two different patients **(A, B)**. Delineations were made on T_2_-weighted scans from the first treatment fraction. The entire prostate is shown in white, the peripheral zone (PZ) in red, and the tumor in yellow. The transition zone (TZ) was extracted in post processing by subtracting the PZ from the prostate delineation. Tumor voxels were excluded from all other zones during analysis.

In two of the patients, no tumor was visible on the diagnostic scans and therefore not delineated. Of one patient with a TURP all remaining tissue was treated as tumor. The median (range) volume of the ROIs were 0.9 (0.1 – 14) cm^3^ for the tumor, 8.9 (5.0 – 26) cm^3^ for the PZ, and 20 (7.2 – 66) cm^3^ for the TZ. An example of the delineations in two different patients is shown in **Figure 1**.

Baseline mean values with the standard error of the mean (SEM) are presented in **Table 3**. These are based on the IVIM scans acquired before the patients received any radiation and the DCE scans acquired directly after a single dose of 3 or 3.1 Gy. One-way ANOVA revealed a statistically significant difference between the tumor, PZ, and TZ for D (*F*_2,44_ = 15, *p* < 0.001), K^trans^ (*F*_2,53_ = 4.3, *p* = 0.02) and v_e_ (*F*_2,53_ = 3.9, *p* = 0.03). No statistically significant correlations were found between IVIM and DCE parameters when using values from the first fraction only.

**Table 3:** Pre-treatment values of the IVIM and DCE parameters.

	Tumor	PZ	TZ
D (10^-3^ s/mm^2^)	1.12 ± 0.08	1.56 ± 0.07	1.45 ± 0.02
f	0.07 ± 0.02	0.09 ± 0.01	0.10 ± 0.01
D^*^ (10^-3^ s/mm^2^)	35 ± 12	28 ± 3	32 ± 2
fD^*^ (10^-3^ s/mm^2^)	3.9 ± 1.3	2.7 ± 0.4	3.2 ± 0.3
K^trans^ (min^-1^)	0.30 ± 0.04	0.14 ± 0.02	0.19 ± 0.02
k_ep_ (min^-1^)	0.58 ± 0.09	0.29 ± 0.07	0.38 ± 0.05
v_e_	0.45 ± 0.08	0.25 ± 0.08	0.44 ± 0.05

The IVIM parameters were acquired before irradiation, the DCE parameters were acquired directly after receiving the first treatment fraction. Mean ± standard error of the mean (SEM) values of all patients are shown.

**Figure 2:**
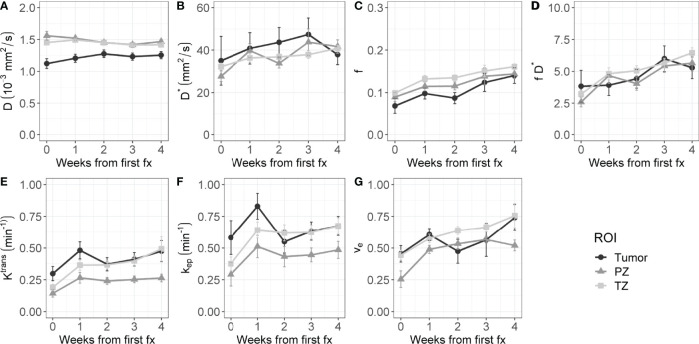
Evolution of intravoxel incoherent motion (IVIM, **A–D**) and dynamic contrast-enhanced (DCE, **E–G**) parameters during radiation treatment. The average value of all patients is shown for the tumor, PZ, and TZ. Error bars indicate the standard error of the mean (SEM).

**Figure 2** shows the average time trends over all patients of the IVIM and DCE parameters. All IVIM and DCE parameters increase in all ROIs over the weeks, except for D in the PZ and TZ. The IVIM perfusion parameters increase steadily over the weeks. The DCE parameters steeply increase from the first to the second week and stabilize or slightly increase after that.

**Figure 3:**
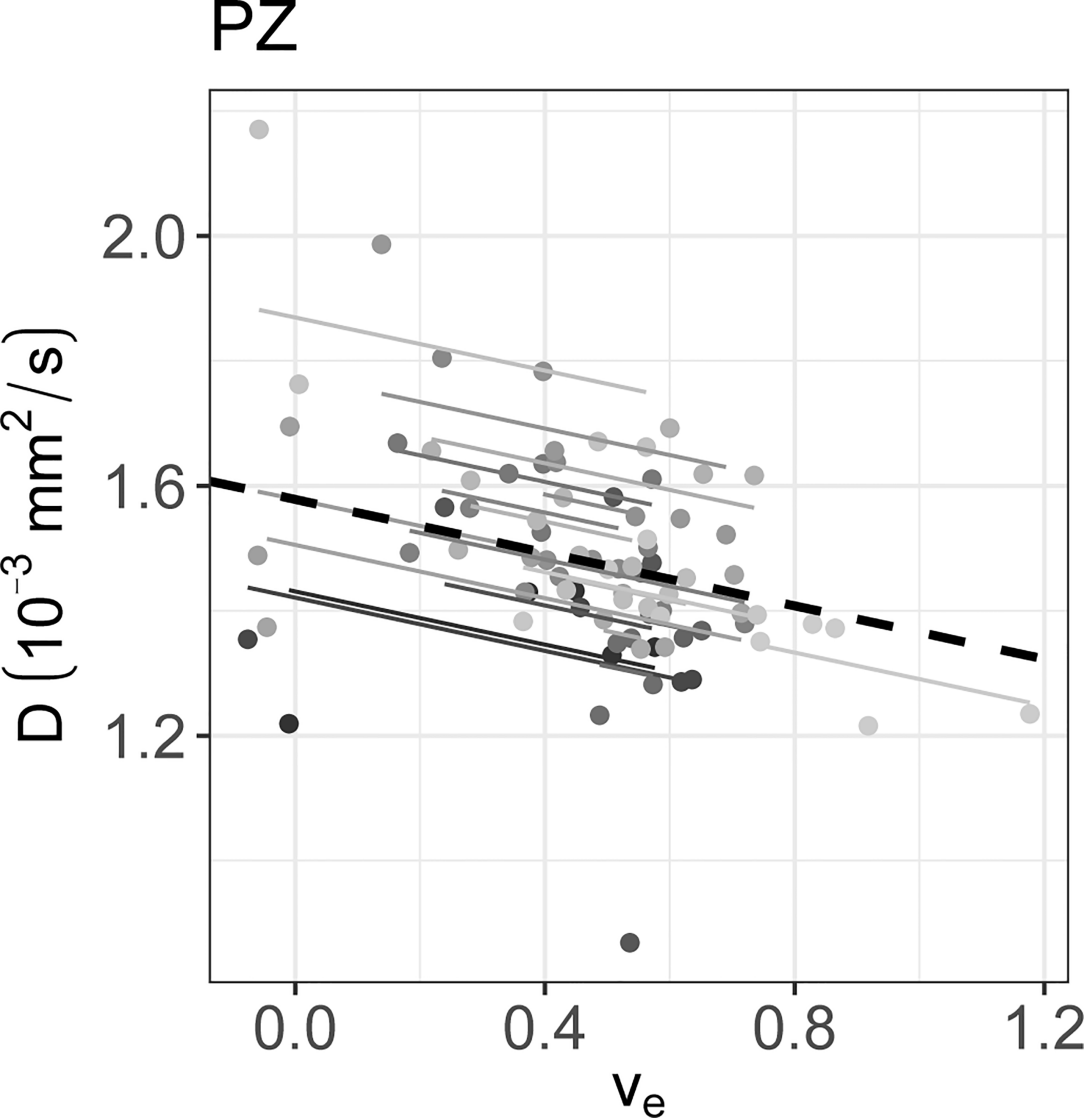
The significant repeated measures correlation of D with the DCE parameters are shown. D only correlated significantly with v_e_ in the PZ. Each line shows the fit for a single patient and the dashed black line shows the overall common slope.

**Figure 4:**
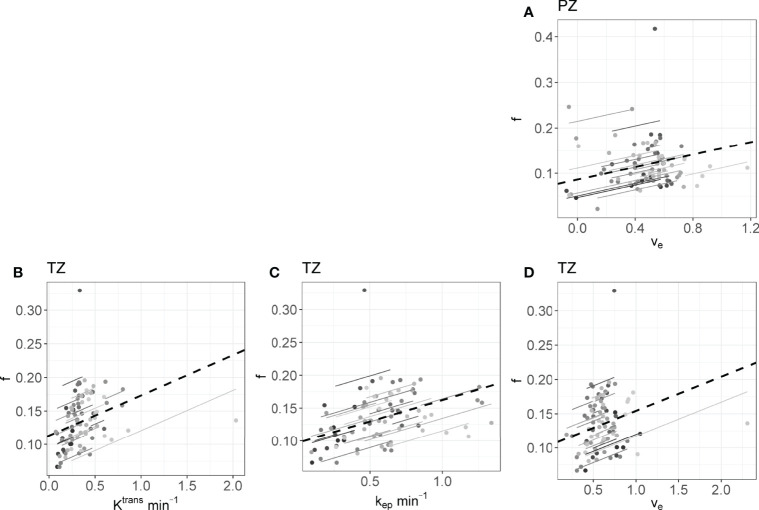
The significant repeated measures correlations of f with the DCE parameters are shown. **(A)** is the correlation between f and v_e_ in the PZ, **(B–D)** are values from the TZ. Each line shows the fit for a single patient and the dashed black line shows the overall common slope.

**Figure 5:**
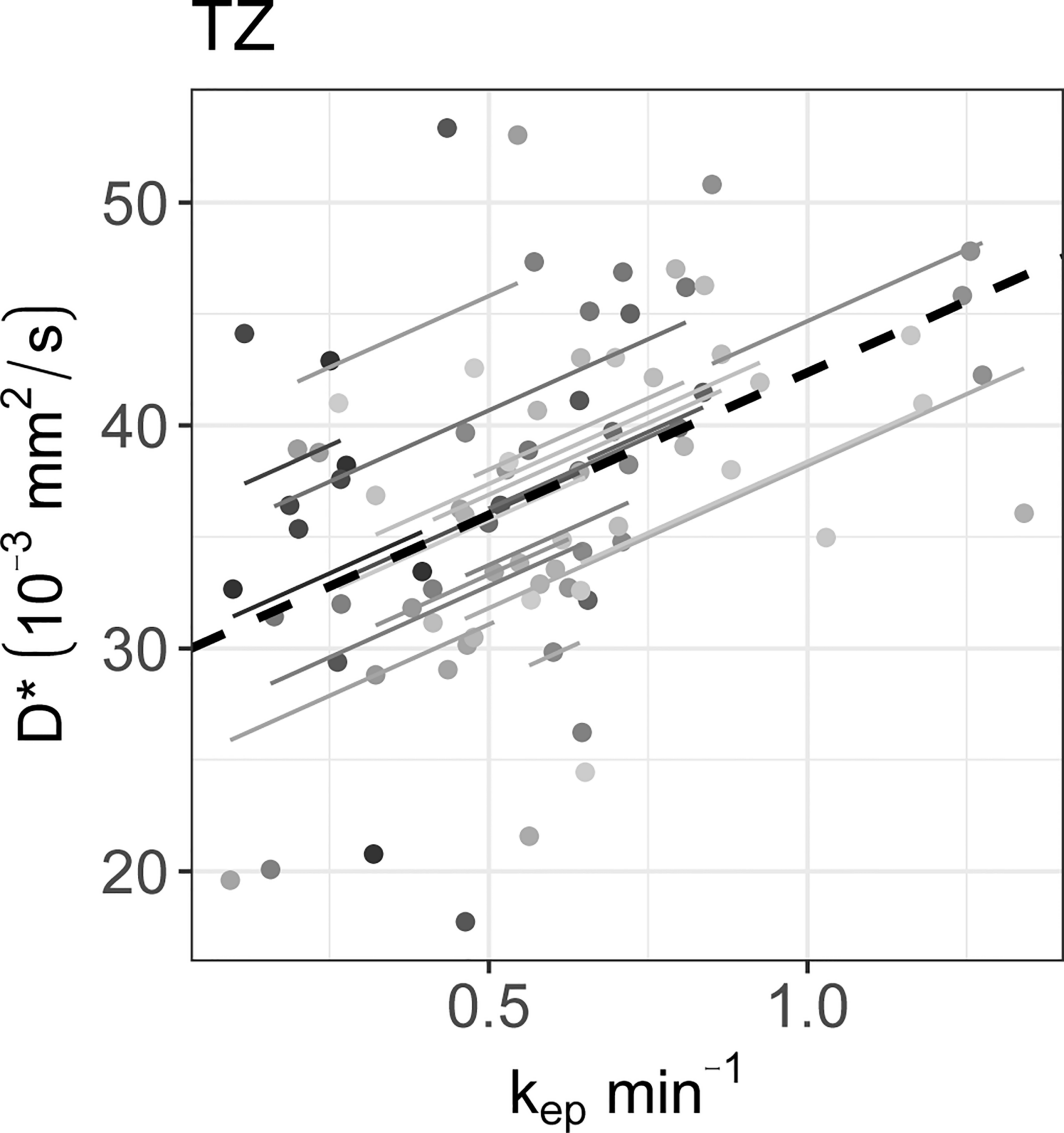
The significant repeated measures correlations of D^*^ with the DCE parameters are shown. D^*^ only correlated significantly with k_ep_ in the TZ. Each line shows the fit for a single patient and the dashed black line shows the overall common slope.

**Figure 6:**
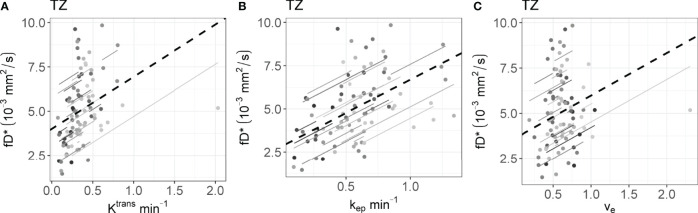
The significant repeated measures correlation of fD^*^ with the DCE parameters are shown. fD^*^ only correlated significantly with DCE parameters in the **(A–C)** TZ. Each line shows the fit for a single patient and the dashed black line shows the overall common slope.

The *r_rm_* calculated on the longitudinal data are presented in **Table 4**. No statistically significant correlations were found between any IVIM and DCE parameter in the tumor. In the PZ, statistically significant correlations were found only between D and v_e_ and between f and v_e_. In the TZ, statistically significant correlations were found between f and K^trans^, f and k_ep_, and f and v_e_. D^*^ correlated significantly only with k_ep_, while the product fD^*^ did so with all DCE parameters. Graphs showing the common slope and the slope per patient of the significant within-subject longitudinal correlations are presented in **Figure 3** for D, **Figure 4** for f, **Figure 5** for D^*^, and **Figure 6** for fD^*^.

**Table 4:** Repeated measures correlations between IVIM and DCE parameters, separately presented for each ROI.

		K^trans^	k_ep_	v_e_
Tumor	D	*r_(60)_* = 0.04 [-0.13, 0.24], p = 0.74	*r_(60)_* = -0.08 [-0.33, 0.19], p = 0.55	*r_(60)_* = 0.19 [-0.05, 0.41], p = 0.15
	f	*r_(60)_* = 0.09 [-0.09, 0.32], p = 0.48	*r_(60)_* = 0.02 [-0.19, 0.27], p = 0.86	*r_(60)_* = -0.12 [-0.43, 0.24], p = 0.34
	D^*^	*r_(54)_* = -0.02 [-0.33, 0.25], p = 0.89	*r_(54)_* = -0.13 [-0.35, 0.13], p = 0.34	*r_(54)_* = 0.02 [-0.23, 0.27], p = 0.90
	fD^*^	*r_(54)_* = 0.03 [-0.24, 0.27], p = 0.82	*r_(54)_* = -0.09 [-0.33, 0.19], p = 0.53	*r_(54)_* = -0.08 [-0.35, 0.22], p = 0.58
				
PZ	D	*r_(63)_* = -0.21 [-0.39, -0.02], p = 0.09	*r_(63)_* = -0.06 [-0.27, 0.15], p = 0.64	***r_(63)_* = -0.33 [-0.54, -0.11], p < 0.01**
	f	*r_(63)_* = 0.21 [0.01, 0.47], p = 0.10	*r_(63)_* = 0.07 [-0.15, 0.39], p = 0.56	***r_(63)_* = 0.33 [0.14, 0.57], p < 0.01**
	D^*^	*r_(63)_* = 0.16 [-0.04, 0.36], p = 0.19	*r_(63)_* = 0.12 [-0.06, 0.32], p = 0.34	*r_(63)_* = 0.04 [-0.16, 0.27], p = 0.75
	fD^*^	*r_(63)_* = 0.23 [-0.03, 0.47], p = 0.07	*r_(63)_* = 0.13 [-0.12, 0.43], p = 0.29	*r_(63)_* = 0.20 [-0.01, 0.40], p = 0.11
				
TZ	D	*r_(63)_* = -0.01 [-0.17, 0.25], p = 0.94	*r_(63)_* = 0.16 [-0.01, 0.35], p = 0.21	*r_(63)_* = -0.13 [-0.29, 0.06], p = 0.29
	f	***r_(63)_* = 0.38 [0.28, 0.64], p < 0.01**	***r_(63)_* = 0.39 [0.19, 0.60], p < 0.01**	***r_(63)_* = 0.37 [0.28, 0.62], p < 0.01**
	D^*^	*r_(63)_* = 0.21 [0.03, 0.52], p = 0.09	***r_(63)_* = 0.35 [0.16, 0.54], p < 0.01**	*r_(63)_* = 0.19 [0.02, 0.53], p = 0.12
	fD^*^	***r_(63)_* = 0.39 [0.26, 0.66], p < 0.01**	***r_(63)_* = 0.48 [0.27, 0.66], p < 0.001**	***r_(63)_* = 0.37 [0.24, 0.63], p < 0.01**

The degrees of freedom are shown between parentheses and the confidence interval of the repeated measures correlation is shown between brackets. Bold values show significant correlations (p < 0.05).

## 4 Discussion

In this study, the longitudinal correlations between IVIM and DCE parameters in different ROIs of prostate cancer patients were assessed during radiation treatment. Weekly IVIM and DCE scans were performed and resulting correlations were tested taking into account the non-independence of repeated measurements on the same patients.

Baseline f and D^*^ values of the IVIM parameters corresponded to values found in the literature, although the reported range is large. The baseline tumor D values found in this study (1.12 ± 0.08 10^-3^ mm^2^/s) were higher than previously found (reported range: 0.13 – 1.06 10^-3^ mm^2^/s) (36). Baseline K^trans^ and v_e_ values were consistent with those found in the literature, while the k_ep_ values were relatively low (37).

When averaging over patients, an increase in all perfusion parameters over the course of radiation treatment can be seen. In the DCE parameter values, this increase was the largest between week 0 and week 1, after which the values seemed to stabilize. This trend is also visible in the IVIM parameters D^*^ and fD^*^. The similar behavior on the group level suggests that there is an overall biological response to radiation that can be measured similarly with both techniques. Previous results comparing DCE parameters before treatment to values acquired at a minimum of two years after treatment showed a decrease in K^trans^ and k_ep_ in the PZ and TZ (38). Taken together with the current results, this could indicate that perfusion is increased during treatment, followed by a decline longer after treatment. The discrepancy between short-term and long-term differences highlights the importance of determining the optimal measurement time for treatment response purposes.

A possible explanation for the early increase of the perfusion parameters in all prostate zones could be an inflammatory response to the radiation treatment in the entire prostate, similar to what was found previously in cervix patients (6). Such an overall response could limit the predictive value of early perfusion for treatment response in prostate cancer patients as it could obscure more subtle changes related to outcome. To investigate this, early changes in perfusion parameters should be related to clinical outcome data. However, these data were not available yet for the current study population.

Although comparison with histology has shown that IVIM parameters provide perfusion information, the specific interpretation of IVIM parameters and their relation to DCE parameters remains unclear (19, 39). Correlations between IVIM and DCE parameters should be carefully interpreted based on the context. When the goal is to assess the ability of both techniques to differentiate between tumor and benign tissue, as done in Pang et al. for prostate cancer (40), it is appropriate to use values from both ROIs combined to determine the correlation. In that case, the correlation reflects how differences between ROIs in IVIM parameters correlate with differences between ROIs in DCE parameters. A ROI effect is clearly visible in the scatterplots presented by Pang et al. (40). However, when investigating longitudinal data, we are interested in the correlation of changes within the ROIs over time. This within-ROI correlation could be different for different ROIs, and theoretically even have an opposite sign compared to the between-ROI correlation. This effect is known as Simpsons paradox (41).

In the current study, the focus is on treatment response monitoring. To measure the longitudinal correlations the *r_rm_* was used on data from each ROI separately. The *r_rm_* can in this case be interpreted for each ROI as the intra-patient correlation between IVIM and DCE parameters while measuring during treatment, indicating the degree to which both parameters reflect the same time trends induced by irradiation.

No significant correlations were found in the tumors. A reason for this could be a low precision of the IVIM and DCE parameters as acquired in the current study. Median values were calculated per ROI, and the variance of these median values scale with 1/n, where n is the number of voxels. As prostate tumors are relatively small, the variance of the median values is relatively high. We showed previously that the test-retest repeatability coefficient of IVIM parameters in prostate tumors is high for the current imaging sequence and analysis: 0.44 10^-3^ mm^2^/s, 0.16 and 76.4 mm^2^/s for D, f, and D^*^ (15). Additionally, DCE parameters are known to have poor repeatability (42–44). Within-patient coefficients of variation reported previously in prostate tumors measured on a 1.5 T system were around 20% for K^trans^, 15% for v_e_ and 30% for k_ep_ (45). Poor repeatability in both IVIM and DCE parameters can attenuate the correlation coefficients (46). In the TZ, which is the ROI with the largest volume, significant positive correlations were found, although all were low (< 0.5).

In order to test the correlations between IVIM and DCE parameters in different ROIs, 36 statistical tests were performed with a significance threshold of *α* = 0.05. This means that the chance of finding at least one false positive result is 84%, because multiple testing inflates the type 1 error rate. However, since this is the first study to test the longitudinal correlation between IVIM and DCE parameters in humans undergoing radiation treatment, type I error rate is less of a concern. These correlations can be used as a direction for future studies.

In conclusion, when assessing changes in group averages over time, a clear increase in IVIM perfusion parameters was found. This increase was also present in all DCE parameters. Although low, it is encouraging that significant longitudinal correlations were found between IVIM- and DCE parameters, suggesting that IVIM could potentially be used as an alternative to DCE for treatment response monitoring purposes, in particular when repeated DCE-MRI is not feasible.

## Data Availability Statement

The raw data supporting the conclusions of this article will be made available by the authors, without undue reservation.

## Ethics Statement

The studies involving human participants were reviewed and approved by the medical ethics committee of The Netherlands; Cancer Institute METC19.1644/N18BREL. The patients/participants provided their written informed consent to participate in this study.

## Author Contributions

EK, PH, MN, FP and UH contributed to the conception and design of the study. VP, MN, FP, and UH contributed to the acquisition of data for the study. EK, PH, and UH contributed to the analysis and interpretation of data for the study. EK wrote the first draft of the manuscript. All authors contributed to manuscript revision, read, and approved the submitted version.

## Funding

This research was funded by ITEA3 project 16016 ‘STARLIT’. UH receives research support from Philips Healthcare and Elekta AB. The funder Elekta AB was not involved in the study design, collection, analysis, interpretation of data, the writing of this article or the decision to submit it for publication.

## Conflict of Interest

The Netherlands Cancer Institute is a member of the Elekta MR-linac consortium, which aims to coordinate international collaborative research relating to the Elekta Unity (MR-linac). Elekta and Philips are commercial partners within the consortium. Elekta financially supports consortium member institutions with research funding and travel costs for consortium meetings.

The authors declare that the research was conducted in the absence of any commercial or financial relationships that could be construed as a potential conflict of interest.

## Publisher’s Note

All claims expressed in this article are solely those of the authors and do not necessarily represent those of their affiliated organizations, or those of the publisher, the editors and the reviewers. Any product that may be evaluated in this article, or claim that may be made by its manufacturer, is not guaranteed or endorsed by the publisher.
