# Supplementary material for: Longitudinal Correlations Between Intravoxel Incoherent Motion (IVIM) and Dynamic Contrast-Enhanced (DCE) MRI During Radiotherapy in Prostate Cancer Patients
Source: Front Oncol. 2022 Jun 7;12:897130. doi: 10.3389/fonc.2022.897130 (PMC9210504; doi:10.3389/fonc.2022.897130)
Supplement: Supplementary file 1 [file DataSheet_1.pdf]

Supplementary Material

1 B1 inhomogeneity

B_1 inhomogeneities influence the conversion of signal intensity to concentration values.[24] To check the variation of the B_1 field, we measured B_1 maps in 3 patients during 5 treatment sessions (four fractions for one patient, and five for the other two).
A dual TR 3D FFE scan was used with a flip angle of 45˚. The first TR was 20 ms, the second 100 ms, and the TE was 2.7 ms. Acquired voxel size was 4 x 4 x 12 mm^3 with a total FOV of 430 x 430 x 60 mm^3. The NSA was set to 3 for a total scan time of 1:29 min.
A consistent pattern was seen in all patients on all fractions with a diagonal band that includes the prostate and left iliac artery, see Figure S1. The B_1 value in this band is close to 100% and drops off to the (diagonal) sides. The iliac arteries were delineated and the median values from the delineations were calculated. The average value of these patients in all fractions was 103 ± 5 % in the left iliac artery and 80 ± 3 % in the right iliac artery. Therefore we only used the AIF from the left iliac artery in this study.

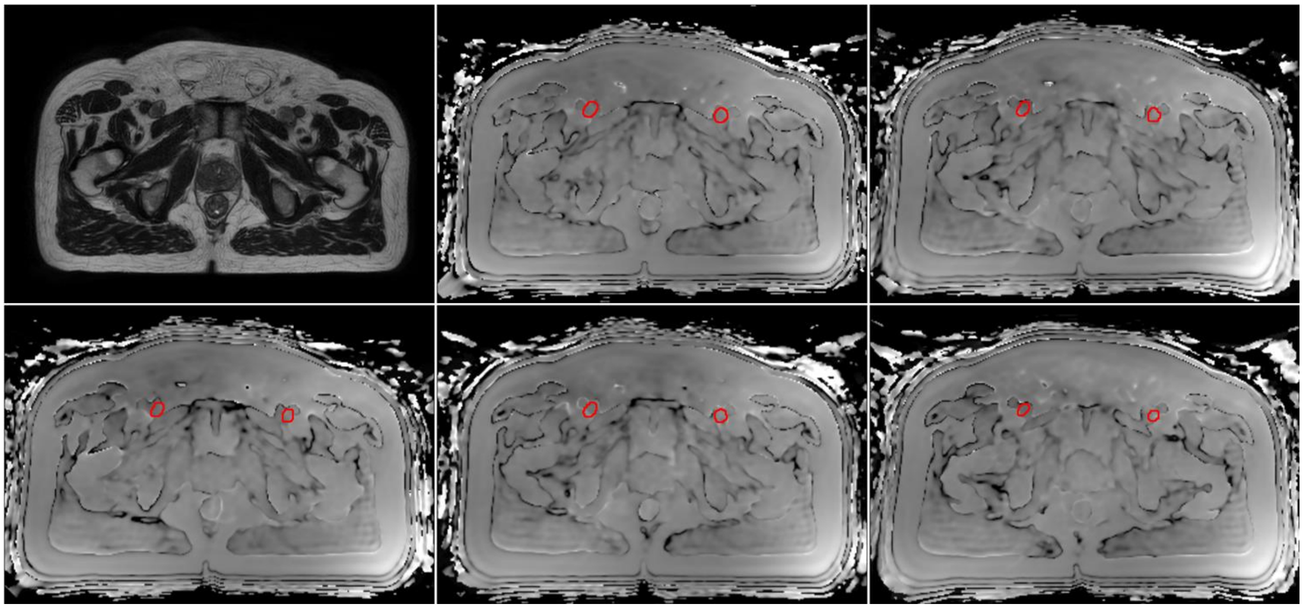

Supplementary Figure 1. B_1 maps of a single patient, acquired during five separate treatment fractions. The delineations show the left and right iliac arteries.

2 Arterial input functions

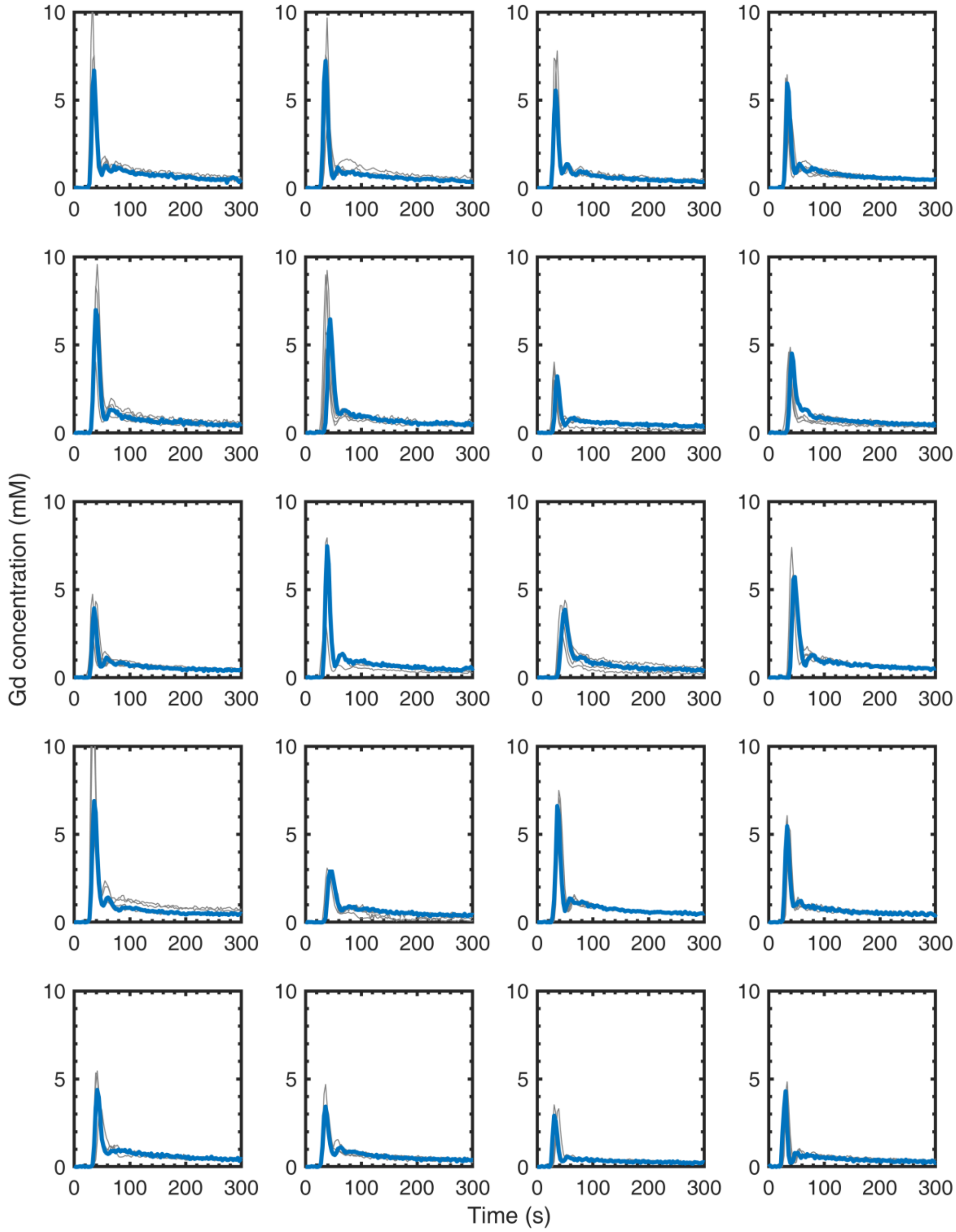

Supplementary Figure 2. Each panel shows the AIFs of a single patient. The AIF was determined for each DCE scan separately (5 scans for 19 patients, 4 scans for 1 patient). The median AIF, which was used for all DCE analyses of that patient, is shown in blue.
